# *Helicobacter pylori*-Related Extraintestinal Manifestations—Myth or Reality

**DOI:** 10.3390/children9091352

**Published:** 2022-09-04

**Authors:** Cristian Dan Mărginean, Cristina Oana Mărginean, Lorena Elena Meliț

**Affiliations:** 1 Department of Pediatrics I, County Emergency Hospital Târgu Mureș, Gheorghe Marinescu Street No. 50, 540136 Târgu Mureș, Romania; 2 Department of Pediatrics I, “George Emil Palade” University of Medicine, Pharmacy, Science, and Technology of Târgu Mureș, Gheorghe Marinescu Street No. 38, 540136 Târgu Mureș, Romania

**Keywords:** *Helicobacter pylori*, extraintestinal manifestations, children, adults

## Abstract

It is well documented that *Helicobacter pylori* (*H. pylori*) can cause both gastrointestinal and extraintestinal manifestations. The latter one represents a major burden in terms of diagnosis and treatment. *H. pylori*-associated systemic subclinical inflammation is mostly responsible for the development of extraintestinal manifestations, and its early eradication might result in preventing all adverse events related to their occurrence. Thus, it was suggested that *H. pylori* might be associated with iron deficiency anemia, thrombocytopenia (immune thrombocytopenic purpura), Schonlein Henoch purpura, failure to thrive, vitamin B12 deficiency, diabetes mellitus, body mass index, cardiovascular diseases, as well as certain neurological conditions. Nevertheless, studies showed both pros and cons in terms of the role of *H. pylori* in the development of previously mentioned clinical entity underlining the crucial need for further studies on these topics. Although most of these extraintestinal manifestations occur during adulthood, we must not forget that *H. pylori* infection is acquired mainly during childhood, and thus its early diagnosis and eradication might represent the cornerstone in the prevention of *H. pylori*-induced inflammatory status and consequently of all related extraintestinal conditions.

## 1. Introduction

The spectrum of disorders caused by *Helicobacter pylori* (*H. pylori*) does not resume to the gastrointestinal tract, resulting in a major burden worldwide in terms of diagnosis and treatment. Thus, aside from the classical damage caused in the host’s stomach resulting in acute or chronic gastritis, peptic ulcer disease, gastric cancer, and gastric-mucosa-associated lymphoid tissue lymphoma, this bacterium has the ability to trigger many other extraintestinal disorders such as iron deficiency anemia, vitamin B12 deficiency, idiopathic thrombocytopenic purpura, or growth retardation [1]. Moreover, recent data suggested a potential relationship between this infection and other less common manifestations such as acute coronary disease, arterial hypertension, diabetes, arterial stiffness in diabetic patients, thyroid disease, glaucoma, stroke, eczema, chronic hives, rosacea, Parkinson’s disease, or Alzheimer disease [1,2]. The most important life-threatening complication of long-term *H. pylori* persistence within the gastric mucosa is represented by gastric carcinogenesis due to the associated chronic inflammation. *H. pylori* infection seems to be involved in the etiology of 80% of all gastric cancers and 5.5% of worldwide malignant conditions [3,4]. Colonization of the gastric mucosa occurs usually during childhood and might be life-long if left untreated. The prevalence of this infection varies in different countries worldwide, especially depending on the socioeconomic status, reaching up to 80% in developing countries or even more [1].

Aside from this dark side of *H. pylori* infection, it was indicated that *H. pylori* might instead have a protective effect against gastroesophageal reflux disease, atopy, allergy and asthma [1]. Therefore, the gram-negative spiral bacterium *H. pylori* might be considered a ‘chameleon’ based on the wide spectrum of disorders that it might cause. *H. pylori*-associated systemic subclinical inflammation seems to be responsible for this wide range of extraintestinal manifestations [2]. In addition, the eradication of this infection was proven to result in the improvement in platelet count and in the efficacy of iron supplementation [5,6]. Although, according to the previously mentioned hypotheses, subclinical inflammation should be related to the long-term persistence of *H. pylori* infection and it should subsequently occur only during adulthood, it seems in fact that the mechanism involved in the development of this inflammatory status does not require a prolonged time since recent studies pointed out the presence of this low-grade inflammation also in children with *H. pylori* infection [7,8]. *H. pylori* virulence factors including flagellin, lipopolysaccharides, cag-pathogenicity island, vacuolating cytotoxin A, pathogen-associated molecular patterns, and adhesins have a major contribution in enabling the long-term persistence of this bacterium and its related harmful effect on the host [9]. The other side of the mechanism involved in the inflammation is represented by the host’s innate and adaptive immune responses, which further trigger the synthesis of a wide range of proinflammatory cytokines and other molecules such as chemokines or chemotactic proteins [10,11]. Innate immunity represented by Toll-like receptors is an important player in the host’s defense mechanisms, but it might also promote *H. pylori* inflammation [12]. 

As we already mentioned, acute *H. pylori* infection usually occurs during childhood, and it might cause unspecific symptoms such as abdominal pain, nausea, vomiting, or even diarrhea, associated with a reduced gastric acid secretion [1]. Although unspecific, epigastric pain seems to be a significant symptom in children with *H. pylori*-positive gastritis [7,13]. The symptoms usually last for a few days, becoming asymptomatic afterwards in most of the cases, but in time, it becomes a chronic process consisting of the infiltration of a high number of immune cells within the gastric mucosa such as mast cells, macrophages, neutrophils, lymphocytes, dendritic cells, natural killer cells, as well as T and B lymphocytes, along with high levels of chemokines and cytokines [14,15]. Furthermore, around 10–20% of individuals diagnosed with *H. pylori* infection eventually develop gastric or duodenal ulcer [16]. In spite of increased incidence of gastric cancer within different countries, it seems that this incidence is strongly correlated with the prevalence of *H. pylori* infection in the same countries [1]. Moreover, effective eradication of this infection was proven to be associated with a significantly reduced risk of gastric cancer [17,18]. 

The first step for effective eradication is in fact represented by accurate diagnosis based on choosing the most appropriate diagnostic method depending on the geographic area, patient’s age, or other *H. pylori*-related peculiarities [19]. Additionally, the diagnosis is even more difficult in patients that present only extraintestinal manifestations triggered by this infection. 

*The aim* of this review was to assess the most important extraintestinal manifestations caused by *H. pylori* in children in order to increase the awareness of practitioners worldwide regarding their relevance for the timely accurate diagnosis of this infection. 

## 2. *H. pylori* and Iron Deficiency Anemia

Iron deficiency represents the most common nutritional deficiency worldwide, affecting at least 500 million people [20]. Taking into account the well-known fact that *H. pylori* is the most common bacterial infection worldwide, it is not surprising that researchers focused on assessing if there is a relationship between them. Blecker et al. described for the first time in 1991 the relationship between *H. pylori* and iron deficiency anemia in a 15-year-old Belgian patient diagnosed with *H. pylori*-induced chronic active hemorrhagic gastritis and iron deficiency anemia, which completely resolved without iron supplementation after *H. pylori* eradication [21]. Several hypotheses were stated in order to explain this relationship such as active hemorrhage from erosive gastritis lesions and anemia associated with chronic inflammation and reduced iron absorption due to achlorhydria [22]. Furthermore, other case reports also sustained this association [22]. Harris et al. assessed the relationship between hypochlorhydria and iron deficiency in a sample of 123 children and noticed that *H. pylori*-positive children associate hypochlorhydria as compared to the uninfected ones sustaining one of the previously mechanisms involved in iron deficiency anemia [23]. Similarly, Soundaravallly et al. [24] compared *H. pylori* schoolchildren with a non-infected one in terms of ferritin levels and pro-oxidant status and concluded that children with *H. pylori* infection have significantly higher levels of malondialdehyde and carbonyls, along with significantly decreased levels of ferritin when compared to *H. pylori* negative group (Table 1). 

As we already mentioned, systemic subclinical inflammatory status triggered by the cytokine storm in the setting of *H. pylori* infection represents the most likely explanation for extraintestinal manifestations. Queiroz et al. [28] also sustained this statement based on their findings, which revealed that interleukin-1β might predict the decreased ferritin and hemoglobin concentrations in children with *H. pylori* infection. Hepcidin is another inflammatory protein which contributes to iron homeostasis by playing an essential role in macrophage iron retention and therefore enables the development of inflammation-associated anemia [52]. Therefore, the second hypotheses that *H. pylori*-induced chronic gastritis might cause *H. pylori* associated iron deficiency anemia was confirmed by Ozkasap et al. [29], who revealed that *H. pylori*-positive children with iron deficiency anemia have significantly higher levels of prohepcidin before eradication. Moreover, a study that compared the serum hepcidin level and the response to oral iron therapy between *H. pylori*-positive and *H. pylori*-negative children proved that serum hepcidin level was associated with a reduced response to the oral iron supplementation in infected children who were associated with iron-deficiency anemia [30]. Other studies also sustained the relationship between *H. pylori* and iron deficiency anemia, as well as the spontaneous resolution of this deficiency after *H. pylori* eradication [26,27]. In terms of eradication, the meta-analysis of Hudak et al. also proved that the eradication regimens added to iron supplementation increased both hemoglobin and ferritin levels [136]. These findings were sustained also by the meta-analyses of Yuan et al. and Huang et al. [137,138] (Table 2). Nevertheless, a recent retrospective study involving 508 subjects diagnosed with *H. pylori* infection and iron deficiency anemia failed in proving the resolution of iron deficiency anemia after *H. pylori* eradication [20]. Similarly, another study performed recently on Indian children with *H. pylori* also stated that *H. pylori* might not, in fact, have an essential role in the development of iron deficiency anemia [36]. Moreover, Emiralioglu N et al. failed in proving a significant difference between prohepcidin level in *H. pylori*-infected children when compared to uninfected ones, even though the mean level was lower in anemic *H. pylori*-positive children [37]. The authors found significantly higher initial levels of prohepcidin, ferritin, and interleukin-6 in infected children, but they noticed no improvement in these parameters after *H. pylori* eradication. Contrarily, another recent study in Egypt pointed out that *H. pylori* infection was more prevalent in patients with refractory or unexplained iron deficiency anemia, revealing also a significant improvement in hematological parameters in patients receiving iron supplements associated with the standard eradication triple therapy in comparison to those receiving only iron supplements [31]. Similar findings were also reported by a study in 2018 which showed that *H. pylori* is involved in iron-deficiency anemia and that the eradication of this infection results in improved blood parameters [32]. Sabbagh et al. also sustained the potential relationship between *H. pylori* infection and iron deficiency anemia, but at the same time stated that *H. pylori* might not be the only one involved in this relationship since poverty, poor nutritional status, and poor treatment might also be a cause for this effect [33]. Furthermore, the results of Rahat A et al. proved that *H. pylori* infection represents a frequent cause of iron deficiency anemia in patients with lower education and females and that 37.5% of *H. pylori*-positive cases presented iron deficiency anemia [25] (Table 1). According to the meta-analysis of Afsar et al., *H. pylori* infection might be associated with adverse pregnancy outcomes, resulting in an increased risk of iron deficiency anemia in pregnant women [139] (Table 2).

Moreover, it was proven that highly virulent *H. pylori* strains such as those expressing cytotoxin-associated gene A or the vacuolating cytotoxin A act via molecular mimicry mechanisms in order to produce or augment iron deficiency [34]. Thus, cytotoxin-associated gene A was suggested to act as a facilitator for *H. pylori* colonization, enhancing iron acquisition and subsequently enabling bacterial survival [35]. International consensus and management guidelines currently recommend that *H. pylori* should be sought and effectively eradicated in patients presenting iron deficiency anemia [34,182]. However not all patients detected with *H. pylori* infection present with iron deficiency anemia, and therefore, further studies should focus on elucidating these controversies [38]. Moreover, based on the most recent reports, several controversies emerged regarding the previously well-documented relationship between *H. pylori* infection and iron deficiency anemia, implying an urgent need for further studies in order to elucidate this mystery (Table 1). 

## 3. *H. pylori* and Purpura

In 1998, Gasbarrini et al. [39] was the first to notice a significant increase in platelet count after *H. pylori* eradication. Furthermore, one year later, Garcia Perez et al. [40] also reported the normalization of platelet count in a patient with chronic immune thrombocytopenic purpura after the eradication of this bacterium. Similarly, Stasi et al. [41] noticed that 50% of adults presented a sustained platelet response following *H. pylori* eradication, especially those with mild forms of immune thrombocytopenic purpura. Thus, studies did not reveal an association between this infection and the severity of immune thrombocytopenic purpura [38]. A more recent study pointed out that patients with immune thrombocytopenia who underwent a successful eradication of *H. pylori* infection were proven to maintain a higher platelet count thereafter [42]. The host response after eradication therapy was indicated to depend on several factors such as the short length of immune thrombocytopenic purpura, age under 65 at the time of immune thrombocytopenic purpura, no prior therapy for this condition, no prior or concomitant steroid therapy, and higher baseline platelet count [41,45,183,184]. Thus, in patients with thrombocytopenia and no bleeding or mild bleeding it is important to avoid treatment as much as possible until the elucidation of the cause. A recent case report of a 57-year-old male which presented petechial rash and gum bleeding increased awareness regarding this recommendation since he was diagnosed with *H. pylori* infection based on stool antigen and presented complete resolution of symptoms and thrombocytopenia following *H. pylori* eradication [43]. Several meta-analyses concluded that *H. pylori* eradication resulted in the increase in platelet count in patients with immune thrombocytopenic purpura [140,141] (Table 2). These findings were also sustained by a review including 11 controlled studies which revealed a platelet count response in 51% of the patients with *H. pylori* infection when compared to 8.8% of *H. pylori*-negative subjects [185]. Therefore, *H. pylori* is a well-documented cause of secondary immune thrombocytopenic purpura since it was proven that the prevalence of this bacterium is higher in patients with this condition as compared to healthy individuals [44]. Multiple mechanisms were involved in the pathogenesis of immune thrombocytopenic purpura in patients diagnosed with *H. pylori* infection such as the induction of platelet aggregated through the von Willebrand triggered by certain *H. pylori* strains, a phenomenon consisting of an activation of monocyte/macrophages with an anti-platelet effect, or molecular mimicry consisting of antibodies formation against *H. pylori* CagA protein and platelet antigens [186] (Table 1).

Further studies also indicated a relationship between these two clinical entities but not all of them encountered statistical significance. Moreover, it seems that the findings depend on the diagnostic method used for detecting *H. pylori* infection. Therefore, serology-based studies found a higher seroprevalence of *H. pylori* infection in patients with immune thrombocytopenic purpura when compared to stool-antigen-based tests [22]. Nevertheless, the results remain controversial since certain stool-antigen-based tests found a considerable higher prevalence in patients with this immune disorders as compared to those without this condition [22]. Aside from the diagnostic method, this relationship was reported to depend on overall *H. pylori* prevalence. Thus, two studies performed in low prevalence areas such as the United States of America and France failed in identifying any link between *H. pylori* infection and immune thrombocytopenic purpura [49,50]. In addition, Mubarak et al. revealed that despite the high prevalence of *H. pylori* infection in pregnant women from Sudan, the study found no association with thrombocytopenia [51]. Surprisingly, *H. pylori* was proven also to have a certain effect also in subjects with a normal level of platelets based on a recent study which pointed out that patients with *H. pylori* infection have a higher mean platelet volume as compared to the *H. pylori*-negative individuals [187]. Based on these findings, the authors hypothesized that in the setting of *H. pylori* infection, the host develops an ongoing mechanism for compensating the destruction process, and certain conditions related to the host or the *H. pylori* strain might be disabled, resulting in immune thrombocytopenia. Nevertheless, pediatric patients were not proven to experience the same effect based on the findings of Săsăran et al., who found no significant difference in terms of mean platelet volume in children with *H. pylori* infection when compared to those with *H. pylori*-negative gastritis or controls [8]. Corroborating the previously mentioned reports in adult patients with these findings in pediatric patients, we might hypothesize that the effect of *H. pylori* on platelets might require a certain amount of time (Table 1).

Based on the aforementioned facts, the detection and eradication of *H. pylori* in patients with immune thrombocytopenic purpura could be extremely useful in clinical practice. Moreover, according to the American Society of Hematology guidelines, eradication therapy should be provided to the patients with immune thrombocytopenic purpura and *H. pylori* infection [188]. The same recommendations were also stated by the European Helicobacter Study Group Consensus and the Second Asia-Pacific Consensus Guidelines [189,190]. Although current guidelines do not recommend screening of all patients with immune thrombocytopenic purpura for *H. pylori* infection, recent studies sustain that this screening would be extremely useful, especially in those originating from areas with a high prevalence [191]. Moreover, based on its ease of administration and limited toxicity, the triple standard eradication regimen with amoxicillin, clarithromycin, and a proton pump inhibitor should be administered to patients with this condition who are detected with *H. pylori* infection in spite of its variable effectiveness [191] (Table 1).

It is worth mentioning that *H. pylori* seems to contribute as well to the pathogenesis of a different type of purpura, Schonlein Henoch purpura, which is known to have an immunological component, but otherwise, individuals have a normal platelet count. Studies in China, where *H. pylori* prevalence is high pointed out that infection is extremely high in children diagnosed with this condition. Thus, it was suggested this type of purpura might also be associated with *H. pylori* infection especially in the setting of gastrointestinal manifestations [46]. The authors concluded that in these endemic areas a screening for *H. pylori* infection would be of great benefit in children with Schonlein Henoch purpura. Furthermore, another study showed that *H. pylori* eradication led either to prompt resolution of Schonlein Henoch purpura or to prevention of recurrences [47]. The meta-analysis of Xiong et al. also pointed out that successful eradication might be associated with a decrease in the recurrence rate in children with Schonlein Henoch purpura [46] (Table 2). A recent review pointed out that the immunological events and local injury of the gastric mucosa triggered by *H. pylori* contribute to the development of Schonlein Henoch purpura [48]. Except for this mechanism, cryoglobulins, elevated serum IgA, C3 levels, proinflammatory molecules, autoimmunity, and molecular mimicry that induce cross-reactive antibodies and immune complexes associated to *H. pylori* infection were also proven to be involved in the course of this condition [48]. Nevertheless, further studies are required to elucidate the complex link between this pathogen and Schonlein Henoch purpura (Table 1).

## 4. *H. pylori* and Growth 

The relationship between *H. pylori* and growth faltering is deeply controversial. This link has been highlighted, especially in countries with poor resources where malnutrition has a considerable high prevalence due to the coexistence in young children between *H. pylori* and other parasitic/enteropathogen infections [52]. Another possible explanation for this fact is that in these areas, also considered endemic area in terms of *H. pylori*, the infection occurs shortly after birth, providing a sufficient amount of time for this bacterium to express this negative effect [192]. Moreover, several meta-analyses which assessed the effect of *H. pylori* infection during pregnancy pointed out a relationship between this infection and gestational diabetes mellitus, preeclampsia, spontaneous abortion, fetal growth restriction, birth defects, and hyperemesis gravidarum [144,145] (Table 2). Jaganath et al. investigated the role of *H. pylori* infection during infancy (6–11 months) and early childhood (12–23 months) in terms of height in children from Peru [61]. The authors noticed that 77% of the included children acquired the infection before 12 months and age most-likely due to the low socioeconomic status, concluding that *H. pylori* was not independently related with growth deficits in these children [61]. Contrarily, other findings sustained the association between *H. pylori* and both growth faltering and malnutrition [53]. More recent studies suggest that mathematical models combining attenuated total reflectance flourier transform infrared spectroscopy and artificial neural networks could be useful in assessing the precise role of this infection in terms of growth failure [193] (Table 1).

The basis of this relationship consists in evidence sustaining that *H. pylori* is related to decreased ghrelin levels, a gastrointestinal hormone responsible for regulating food intake. Thus, a study including 50 children proved that gastric ghrelin levels returned to normal once the infection was successfully eradicated, but body mass index showed no significant differences [54]. Other studies also pointed out this relationship [62,194]. Aside from ghrelin, certain authors found a low level of leptin in *H. pylori*-infected children, which improved following eradication [55,56]. The consequences of these pathological findings had a negative effect on growth, but they were also proven to result in short stature and recurrent infection due to decreased immunity [195,196]. A more complex study from the Czech Republic that assessed vital signs and body parameters in *H. pylori*-positive versus *H. pylori*-negative subjects with an overall *H. pylori* prevalence of 5.2% concluded that this infection was associated with short stature in children, but not with body weight, body mass index, and blood pressure either in children and adolescents or in adults [57]. Similarly, a review assessing studies which included children from low- and middle-income areas suggested a potential relationship between *H. pylori* and growth retardation but concluded that there is not sufficiently strong evidence to justify screening in order to prevent this consequence [58]. Furthermore, Chiu et al., performing a study on a large sample of children, found no association between failure to thrive and *H. pylori* association [197]. 

Recent studies underlined the role of gastrointestinal microflora, including *H. pylori,* in terms of molecular mimicry representing antigens sources, which resemble appetite-regulating peptides [59]. Moreover, certain common sequences were identified between leptin and the intestinal microflora proteins of *Lactococcus lactis*, *Lactobacillus bacteriophage*, *Escherichia coli*, *Candida*, and *Aspergillus* [59]. Thus, experimental studies on serum samples from children with growth hormone deficiency and short stature pointed out that certain children that were infected with *H. pylori* as well as those exposed to Candida albicans present antibodies against leptin, ghrelin, orexin A, and α-MSH, with a potentially negative effect on the physiological functions of these molecules [53,60]. However, based on these multiple controversies, further studies on larger samples should definitely be performed in order to elucidate all mechanisms involved in the relationship between *H. pylori* and growth retardation in children (Table 1). 

## 5. *H. pylori* and Vitamin B12 Deficiency 

*H. pylori*-induced gastritis was proven to result in a functional inhibition of parietal cells causing hypochlorhydria. The increase in gastric pH will lead to the malabsorption of several vitamins and other minerals [79]. 

The relationship between vitamin B12 deficiency and *H. pylori* infection was reported for the first time in 1984 by O’Connor et al., who identified Campylobacter-like organisms in patients with type A gastritis associated with pernicious anemia [63]. Several studies proved a link between this infection and the malabsorption of vitamin B12, pointing out that this deficiency was present in more than half (67.4%) of the patients with *H. pylori* infection [64,65]. Moreover, even in the setting of normal serum vitamin B12 levels, studies revealed a higher prevalence of this infection in patients with these levels at the lower end of the normal range [66]. Tsay et al. suggested that studies to follow the effect of eradication therapy on vitamin B12 level would be useful for clearly determining the role of *H. pylori* infection on vitamin B12 status [198]. Nevertheless, a case report of a 35-yearl-old male with *H. pylori* infection and vitamin B12 deficiency pointed out that the vitamin B12 level normalized after 1 month of vitamin B12 supplementation and standard triple therapy for *H. pylori* eradication [67]. In fact, vitamin B12 deficiency is very common worldwide, accounting for 20–60% of incidences in developing countries and up to 20% in developed ones [32]. Thus, a recent study performed on adults with *H. pylori* infection showed a significant association between the presence of this infection and vitamin B12 deficiency [32]. Moreover, Annibale et al. [73] found *H. pylori* to be the only pathological elements in 57.1% of patients with macrocytic anemia due to vitamin B12 deficiency (Table 1).

Another potential mechanism for this deficiency might be related to the treatment with antacid drugs [19]. Furthermore, *H. pylori* might play the role of molecular mimicker since *H. pylori* expresses an antigen which is similar to H^+^/K^+^-adenosine triphosphate protein [199]. Eventually, vitamin B12 deficiency leads to hyperhomocysteinemia, which might be a risk factor for both cerebrovascular and ischemic heart diseases, therefore linking *H. pylori* infection and vascular disorders [68]. Based on all these findings, the Maastricht IV/Florence Consensus 2012 included unexplained vitamin B12 deficiency in the management guide of *H. pylori* infection [200]. Based on the major relevance of this vitamin deficiency, further studies to assess the role of *H. pylori* infection in its occurrence could result in the development of further effective preventive strategies with major impact on also reducing subsequent cardiovascular events (Table 1).

## 6. *H. pylori* and Cardiovascular Diseases

Cardiovascular diseases include coronary artery disease, stroke and peripheral artery disease with increased rates of morbidity and mortality worldwide. Studies pointed out that the presence of *H. pylori* at the level of carotid plaques might result in their instability and eventually lead to ischemic stroke, especially in patients infected with CagA gene positive strains [69]. The authors also underlined an association between *H. pylori* and acute cerebral ischemia in patients with ischemic cerebrovascular stroke. These findings were also sustained by the meta-analysis of Doheim et al., who emphasized the significant association between *H. pylori* infection and the increased risk of stroke [149]. Nevertheless, Yu et al. found no association even in those infected with Cag-A positive strains [150] (Table 2). Furthermore, a study from Korea pointed out a positive association between this infection and low HDL, elevated LDL, and cardiovascular disease, reporting that eradication of this bacteria lowered the risk of high LDL and low HDL but had no effect on cardiovascular disease [72]. These findings were sustained by a very recent review, which concluded that *H. pylori* infection increased the risk of adverse cardiovascular events by 51%, especially in terms of myocardial infarction and cerebrovascular disease [70]. Therefore, clarithromycin-based eradication therapy might lower mortality rate in patients at risk, such as those with arterial hypertension [201]. Two recent meta-analyses revealed that *H. pylori* infection was positively associated with arterial hypertension representing an essential factor in the development of this condition [76,151] (Table 2). Nevertheless, another study from Japan found no relationship between *H. pylori* infection and either stroke mortality risk or coronary heart disease [202] (Table 1). The results regarding the effect of *H. pylori* on coronary heart disease remain controversial because, while several studies sustained an association between these two pathologies [203,204,205,206], other studies failed in identifying any association [207,208,209,210]. A concerning fact is that according to the meta-analysis of Rahmani et al., most of the patients with coronary heart disease acquired the infection during childhood [152]. Another meta-analysis also pointed out that *H. pylori* infection might augment the risk of coronary heart disease during early life [153]. Coronary instability and acute coronary syndrome might also occur as a result of *H. pylori* infection [154,155] (Table 2). Moreover, it was suggested that *H. pylori* eradication might result in an improvement in endothelial function [211], but the findings remain inconsistent [212]. *H. pylori*-associated inflammation plays a major role in the development of atherosclerosis based on the wide spectrum of pro-inflammatory cytokines associated with this infection [213]. Several meta-analyses pointed out a significant positive association between *H. pylori* infection and the risk of atherosclerosis, proving that the infection has the ability to promote atherosclerosis development in people below the age of 60 years but also in those without cardiovascular risk factors [146,147,148] (Table 2). In addition, this systemic inflammation induces the synthesis of acute phase coagulation proteins such as fibrinogen, which is particularly important in the development of coronary heart disease [214], thus also suggesting a possible link between *H. pylori* infection and other thrombotic events, including stroke. Another possible explanation for the relationship between *H. pylori* infection and coronary heart disease might be related to the ability of this infection to induce platelet aggregation, resulting in instability of atherosclerotic lesions [215]. CagA virulent strains are associated with an increased risk of thrombotic events [213]. Moreover, these strains were reported to play a major role in destabilizing coronary plaques, resulting in acute coronary syndromes [154]. Furthermore, Rahmani et al. highlighted in their meta-analysis a significant association between *H. pylori* infection and myocardial infarction [158] (Table 2). Although in the meta-analysis of Yan et al., the authors suggested a possible relationship between *H. pylori* infection and arrhythmia, including atrial fibrillation [156], the results are rather inconsistent [157]. Nevertheless, *H. pylori* eradication was proven to decrease the incidence of arrhythmia in Asian and African subjects [156] (Table 2).

In terms of risk factors, Kim et al. pointed out that *H. pylori* infection was a significant and independent predictor or dyslipidemia [71]. Mladenova et al. suggested that host’s genetic susceptibility might represent the response to the question why only certain individuals with *H. pylori* infection develop adverse cardiovascular events [216]. Based on these controversial reports, and the worldwide mortality due to cardiovascular events, further studies are definitely required in order to clearly determine the role of this infection in the development of cardiovascular diseases. 

## 7. *H. pylori* Infection, Insulin Resistance, Diabetes Mellitus, and Obesity

*H. pylori* seems to also be related with insulin resistance, diabetes mellitus, and metabolic syndrome [79]. Nevertheless, while certain studies sustained a higher prevalence of *H. pylori* in patients with diabetes mellitus, others failed in identifying any association between these two [89,90,91]. Thus, Nasif WA et al. proved that *H. pylori* prevalence was more increased in type 2 diabetes mellitus patients as compared to non-diabetic individuals [75]. Eradication of *H. pylori* infection was proven to decrease the risk of diabetes [77]. Additionally, Song et al. recently pointed out that diabetic patients with *H. pylori* infection might require more aggressive eradication therapies, especially those with poor glycemic control and greater body mass index [78]. Therefore, Mansori et al. concluded in a recent meta-analysis that *H. pylori* eradication should be considered in patients with diabetes mellitus [160]. Nonetheless, Upala et al. found no improvement in insulin resistance, fasting blood glucose, and metabolism paraments after eradication of *H. pylori* [159] (Table 2). A more recent study also indicated a significantly higher prevalence of *H. pylori* infection in patients with diabetes as compared to controls with a more increased chance to be symptomatic [74]. *H. pylori* infection might also increase the risk of diabetes-related complications, being associated with proteinuria [162] (Table 2). Moreover, it was suggested that there is a relationship between the presence of this infection and insulin resistance in normal-weight subjects [79]. Studies emphasized once more that *H. pylori*-associated inflammation and subsequent production of cytokines along with hormonal imbalances are responsible for the association between this infection and diabetes mellitus [217] (Table 1).

In diabetic patients, the risk of atherosclerotic vascular disease seems to be linked to the increased levels of serum oxidized low-density lipoprotein associated with *H. pylori* infection [75]. Furthermore, obesity was not proven to enhance insulin resistance in the setting of *H. pylori* infection, it being stated that *H. pylori* is in fact responsible for adverse lipid profile outcomes [92]. In terms of lipid profile parameters, *H. pylori* eradication was proven to increase the high-density lipoprotein levels [166] (Table 2). However, it was emphasized that the *H. pylori* eradication rate is lower in obese patients when compared to controls [80]. Similarly, Hamrah et al. pointed out a significant association between *H. pylori* infection and both diabetes mellitus and increased body mass index in patients from Afghanistan [82]. In terms of obesity, most of the reported meta-analyses sustain a significant positive association between *H. pylori* and obesity risk [159,165,167] (Table 2). Moreover, a recent study from Douala-Cameron showed that *H. pylori* and high body mass index, separately or not, were proven to be risk factors for diabetes mellitus [81]. Contrarily, Alzahrani et al. found a relationship only between *H. pylori* infection and increased body mass index and not with type 2 diabetes mellitus [83]. Likewise, a recent study from China also reported that *H. pylori* was not significantly associated with diabetes [93]. Alzahrani et al. also failed in identifying any association between *H. pylori* seropositivity and the risk of developing diabetes even in adults at high risk for this condition [94]. However, even the results in the same population remain controversial since another study from China revealed that *H. pylori* is significantly associated associated with diabetes [84] (Table 1).

It seems there is an interdependence relationship between *H. pylori* infection and diabetes since it was proven that not does only *H. pylori* influence diabetic patients, but diabetes also influences the severity and localization of gastric inflammation induced by *H. pylori* infection. Yang et al. proved that the severity of corpus gastritis associated to *H. pylori* infection was more severe in type 2 diabetes mellitus patients when compared to non-diabetic controls [85]. In addition, the authors highlighted that non-insulin users and male gender presented a higher risk for developing corpus-predominant gastritis following *H. pylori* infection. Therefore, diabetes mellitus and *H. pylori* infection are two highly prevalent conditions which may share certain common pathogenetic mechanisms affecting each other and may coexist beyond the simple coincidence [87], but further studies are required on different populations in order to elucidate that question (Table 1). 

In terms of pediatric age, the evidence is scarce. However, a recent study found no significant association between diabetes and *H. pylori* infection in children aged between 5 and 15 years [95]. Moreover, the study pointed out no difference in glycemic control between type 1 diabetes mellitus children with or without *H. pylori* infection. A potential explanation might be related to the insufficient amount of time required for *H. pylori* to trigger its related systemic complications. 

Non-alcoholic fatty liver disease (NAFLD) was recently linked to *H. pylori* infection [198]. Thus, Kim et al. pointed out that subjects infected with *H. pylori* had a higher incidence of NAFLD as compared to uninfected controls [86]. Similar findings were reported by Polyzos, who noticed that NAFLD patients had higher circulating levels of anti-*H. pylori* IgG [87]. A recent meta-analysis performed by Wijarnpreecha et al. also pointed that patients detected with *H. pylori* infection have a higher risk of NAFLD [88]. Contrarily, studies from Japan and Korea stated exactly the opposite [96,97] (Table 1).

## 8. *H. pylori* and Neurological Conditions

Recent evidence pointed out a potential link between *H. pylori* infection and certain neurological conditions such as Parkinson’s disease, multiple sclerosis, or Alzheimer’s disease, but the results remain controversial. 

It was suggested that *H. pylori* might damage dopaminergic cells in central nervous system contributing to the development of Parkinson’s disease [98]. Furthermore, Tan et al. pointed out that *H. pylori* might worsen motor severity in patients with Parkinson’s disease [99]. In terms of eradication effects, studies are even more controversial. Thus, certain researchers sustain that *H. pylori* eradication was associated with an improvement in levodopa action, clinical symptoms, and life quality in patients with Parkinson’s disease [20], while others failed to find any association between bacterial eradication and clinical outcomes of Parkinson’s disease [110]. A recent review pointed out that the host’s innate immunity represented by toll-like receptor 2 might represent the underlying link between this neurological condition and *H. pylori* infection [100] (Table 1). 

In terms of multiple sclerosis, it was highlighted that *H. pylori* infection was commonly encountered in this group of patients [101]. However, the findings reported in the literature remain conflicting since Long et al. [102] pointed out an increased prevalence of *H. pylori* infection in patients with multiple sclerosis, while Yaoa et al. found a negative correlation between these two conditions [111]. Oppositely, Gerges et al. found a significantly higher *H. pylori* seropositivity in Egyptian multiple sclerosis patients, especially those with secondary progressive forms [103]. Moreover, Baj et al. also reported both harmful and protective effects of *H. pylori* infection on multiple sclerosis [100] (Table 1).

The neurodegenerative diseases called Alzheimer’s disease was related to certain bacterial or viral pathogens such as *H. pylori*, *Chlamydia pneumonia*, or *Herpes simplex virus-1* [104]. Thus, *H. pylori* infection was found to increase the risk of Alzheimer’s disease, while its eradication was associated with improvement in Alzheimer’s disease symptoms [106,107]. Other studies found significantly higher levels of anti-*H. pylori* IgG antibodies in both serum and cerebrospinal fluid of Alzheimer’s disease patients when compared to controls [108]. Moreover, those with increased seropositivity experienced a poorer clinical outcome. A recent systematic review also pointed out a possible association between this neurodegenerative condition and gastrointestinal microbiota, including *H. pylori* [109]. Thus, bacteria were proven to contribute to neurodegeneration by promoting inflammation, molecular mimicry mechanisms and accumulation of amyloid beta into the brain [218]. Therefore, gastrointestinal disorders, including *H. pylori* might negatively impact Alzheimer’s disease development and clinical course [105], but further studies are definitely required (Table 1).

The results of several meta-analyses regarding the role of *H. pylori* infection and Parkinson’s or Alzheimer’s disease remain controversial [105,170,171,172,173] (Table 2). 

## 9. *H. pylori* and Other Extraintestinal Manifestations

### 9.1. Dermatological Conditions

Rosacea, a chronic facial dermatitis manifesting as erythema and cutaneous lesions characterized by very dilated red superficial capillaries, also known as teleangiectasia, is the most common dermatological condition associated with *H. pylori* infection [112]. Although several studies have indicated a potential relationship between this infection and rosacea, the precise role of *H. pylori* in the pathogenesis of rosacea remains debatable. Thus, it was proven that *H. pylori* infection is significantly more common in patients with rosacea when compared to control group [219]. 

*H. pylori* infection, but not small intestinal bacterial overgrowth, may play a pathogenic role in rosacea [219]. Moreover, the authors proved that the eradication of this infection was associated with either the improvement or total resolution of rosacea cutaneous lesions in 96.9% of the patients. Similar findings were also reported by other studies on this topic [113,114]. 

Chronic urticaria or itchy rash consisting of wheal-like lesions was also reported to be associated with *H. pylori* infection [115,116]. Contrarily, other authors failed in identifying a relationship between these two conditions; still, they pointed out a positive effect of eradication therapy on skin lesions [117]. 

Autoimmune bullous diseases consisting of pemphigus, pemphigoid, dermatitis herpetiformis, epidermolysis bullosa acquisita, and linear immunoglobulin A disease were also suggested to be associated with *H. pylori* infection [112]. The scarce evidence reported on this topic proved a higher level of IgG anti-*H. pylori* antibodies in patients with these conditions, revealing a higher prevalence of *H. pylori* infection in patients with autoimmune bullous diseases [220,221]. 

Data regarding the association between *H. pylori* infection and psoriasis or alopecia aerata remain contradictory, calling for further studies on larger samples [112].

### 9.2. Ophthalmic Disease 

Recent evidence pointed out a potential relationship between open-angle glaucoma, a condition that might result in optic nerve damage, and *H. pylori* infection [118]. A recent meta-analysis performed on 10 studies proved that the prevalence of *H. pylori* infection was twice as high in patients with this ophthalmic condition as compared to a control group, revealing at the same time a normalization of mean visual field parameters and intraocular pressure as a result of *H. pylori* eradication [119]. 

Another ophthalmic condition, central serous chorioretinopathy, which might lead to micropsia, decreased vision acuity, metamorphopsia, and dyschromatopsia, was also suggested to be associated with *H. pylori* infection [118]. Based on the findings of Cotticelli et al., *H. pylori* infection prevalence was twice as high in patients with this condition as compared to controls [222]. Other authors highlighted both an improvement of central serous chorioretinopathy and a reduction in the recurrence rate following *H. pylori* eradication [120,121]. 

### 9.3. Autoimmune Conditions

The relationship between rheumatoid arthritis and *H. pylori* infection raised several controversies. Studies in vitro pointed out that B cells chronically stimulated with *H. pylori*-produced urease resulted in the production of autoantibodies, including IgM rheumatoid factor [122]. Nevertheless, studies regarding the prevalence of *H. pylori* in patients with this autoimmune condition failed in proving a significant difference in prevalence rate when compared to healthy controls [15,132]. In fact, studies on this topic revealed a similar prevalence between rheumatoid arthritis patients and healthy controls [223]. The eradication effect on rheumatoid arthritis evolution also remains debatable since both positive [128,129] and null [130,131] findings were reported. The risk of rheumatoid arthritis and lupus erythematosus in the setting of *H. pylori* infection might be related to the virulence features of this bacterium [123]. 

In terms of systemic lupus erythematosus, studies on mice proved that exposure to the urease produced by *H. pylori* might lead to the synthesis of anti-double-stranded DNA antibodies [122]. The contradictions go further since other authors suggested that *H. pylori* might represent a protective factor against systemic lupus erythematosus development in African-American females [133]. A more recent study performed in Taiwan revealed a 1.63-fold higher risk of systemic lupus erythematosus in infected females below the age of 30 years [124]. Furthermore, it is well-documented that *H. pylori* infection triggers a Th-17 inflammatory response, which is also involved in the pathophysiology of systemic lupus erythematosus [224,225]. In fact, a recent review concluded that *H. pylori* might have a dichotomous role acting as both a trigger and a protector for systemic lupus erythematosus depending on age, race, and the affected organs [125]. 

Sjögren syndrome, a systemic autoimmune disease characterized by lymphoplasmocytic infiltration of the exocrine glands resulting in sicca syndrome was also suggested to be associated with *H. pylori* infection [125]. *H. pylori* antibodies were found to be significantly increased in patients with primary Sjögren syndrome as compared to secondary type, other autoimmune diseases, and healthy controls [126]. Similar findings were reported by other authors [226,227,228]. Nevertheless, the benefit of *H. pylori* eradication in patients with this autoimmune condition remains controversial [134]. 

Regarding systemic sclerosis, the role of *H. pylori* infection follows the same pattern of controversies [223]. *H. pylori* seropositivity is not uncommon in patients with systemic sclerosis [127]. Nevertheless, other studies failed in identifying a significant difference regarding *H. pylori* prevalence in systemic sclerosis patients and healthy controls but revealed that almost all patients with systemic sclerosis were infected with more virulent *H. pylori* strains, especially those expressing CagA [135]. Contrarily, Kalabay et al. found an increased prevalence of *H. pylori* infection in patients with systemic sclerosis [229]. The same authors suggested a potential relationship between *H. pylori* infection and increased severity of systemic sclerosis. Therefore, *H. pylori* might be involved in the activity of systemic sclerosis [223].

## 10. Concluding Remarks

Emerging evidence suggests more and more extraintestinal pathologies to be related with *H. pylori* infection. Although most of them do not occur during childhood, acknowledging their existence is crucial for eradicating *H. pylori* infection in children since it is well-documented that *H. pylori*-associated inflammation is initiated in early life. Therefore, preventing the development of all aforementioned extraintestinal manifestations is possible only in the setting of early diagnosis and eradication of this infection. Moreover, it seems that the development of extraintestinal manifestations as a result of *H. pylori* infection might depend also on other factors such as age, race, gender, and even geographical areas. Therefore, future perspectives should also focus on elucidating the role of these demographic and host-related factors in the pathogenesis of *H. pylori*-associated extraintestinal manifestations. Likewise, a more in-depth study of host-related inflammatory response as a result of *H. pylori* infection should be performed for identifying potential inflammatory pathways that might be used for designing targeted therapies in order to prevent further complications related to this infection, including extraintestinal manifestations. It would add a great value if these studies were performed on pediatric patients for increasing the effectiveness of potential therapies that could be developed.

Indeed, pros and cons exist regarding the involvement of *H. pylori* in each of these extraintestinal manifestations imposing further studies in order to identify all the mechanisms related to the development of this conditions in which this bacterium might be implicated.

## Figures and Tables

**Table 1:** *H. pylori* and extraintestinal manifestations—Pros and Cons.

Diseases	Pros *H. pylori*	Cons *H. pylori*
**Iron deficiency anemia**	*H. pylori* infection represents a frequent cause of iron deficiency [25] completely resolved without iron supplementation after *H. pylori* eradication [21,26,27]. interleukin-1β—the decreased ferritin and hemoglobin concentrations in children with *H. pylori* infection [28] *H. pylori*-positive children with iron deficiency anemia have significantly higher levels of prohepcidin before eradication [29] serum hepcidin level associated with a reduced response to the oral iron supplementation in infected children [30] *H. pylori* infection more prevalent in patients with refractory or unexplained iron deficiency anemia → significant improvement in hematological parameters in patients receiving iron supplements associated with the standard eradication triple therapy [31] eradication improves blood parameters [32] Sabbagh et al. → potential relationship between *H. pylori* infection and iron deficiency anemia, but at the same time stated that *H. pylori* might not be the only one involved in this relationship [33] highly virulent *H. pylori* strains produce or augment iron deficiency [34] Cag A → facilitator for *H. pylori* colonization enhancing iron acquisition [35]	Tseng et al. → failed in proving the resolution of iron deficiency anemia after *H. pylori* eradication [20] Indian study on children → *H. pylori* might not, in fact, have an essential role in the development of iron deficiency anemia [36] Emiralioglu N et al. → no difference between prohepcidin level in *H. pylori* infected children when compared to uninfected ones [37] not all patients detected with *H. pylori* infection associate iron deficiency anemia [38]
**Purpura**	significant increase in platelet count after *H. pylori* eradication [39] Garcia Perez et al. [40] → normalization of platelet count in a patient with chronic immune thrombocytopenic purpura after the eradication of the bacterium 50% of adults—a sustained platelet response following *H. pylori* eradication [41] patients with immune thrombocytopenia who underwent a successful eradication of *H. pylori* → maintain a higher platelet count thereafter [42] complete resolution of symptoms and thrombocytopenia following *H. pylori* eradication [43] *H. pylori* → cause of secondary immune thrombocytopenic purpura [44] the pathogenesis of immune thrombocytopenic purpura → induction of platelet aggregated through the von Willebrand triggered by certain *H. pylori* strains → an activation of monocyte/macrophages with an anti-platelet effect, or antibodies against *H. pylori* CagA protein and platelet antigens [45] Schonlein Henoch purpura associated with *H. pylori* infection, especially in the setting of gastrointestinal manifestations [46] *H. pylori* eradication led either to prompt resolution of Schonlein Henoch purpura or to prevention of recurrences [47] local injury of the gastric mucosa triggered by *H. pylori* contribute to the development of Schonlein Henoch purpura [48]	two studies from United States of America and France → no link between *H. pylori* infection and immune thrombocytopenic purpura [49,50] other studies no association between this infection and the severity of immune thrombocytopenic purpura [38] Sudan study → no association between *H. pylori* infection and thrombocytopenia in pregnant women [51] Săsăran et al. → on pediatric patients → no significant difference in terms of mean platelet volume [8]
**Growth**	link between growth faltering and *H. pylori* → in countries with poor resources [52] correlation between *H. pylori* and both growth faltering and malnutrition [53] gastric ghrelin levels returned to normal once the infection was successfully eradicated [54] low level of leptin in *H. pylori* infected children—improved following eradication [55,56] Czech study → *H. pylori* infection was associated with short stature in children [57] low- and middle-income countries → potential relationship between *H. pylori* and growth retardation [58] gastrointestinal microflora including *H. pylori*—molecular mimicry [59] children infected with *H. pylori*—antibodies against, leptin, ghrelin, orexin A, and α-MSH [53,60]	A study in Peru → 77% of the children which acquired the infection before 12 months of age with low socioeconomic status → *H. pylori* was not independently related with growth deficits in these children [61] body mass index showed no significant differences after eradication [54] Chiu et al.—no association between failure to thrive and *H. pylori* [62]
**Vitamin B_12_ deficiency**	in adults with *H. pylori* infection → significant association between the presence of this infection and vitamin B12 deficiency [32] O’Connor et al. → vitamin B12 deficiency and *H. pylori* infection [63] this deficiency present in > a half (67.4%) of the patients with *H. pylori* infection [64,65] a higher prevalence of *H. pylori* infection in patients with vitamin B12 levels at the lower end of the normal range [66] vitamin B12 level normalized after 1 month of vitamin B12 supplementation and standard triple therapy [67] Annibale et al. [60] → *H. pylori* the only pathological elements in 57.1% of patients with anemia due to vitamin B12 deficiency vitamin B12 deficiency—link between *H. pylori* infection and vascular disorders [68]	
**Cardiovascular diseases**	*H. pylori*—carotid plaques instability => ischemic stroke [69] association between *H. pylori* and acute cerebral ischemia in patients with ischemic cerebrovascular stroke [69] Korea → a positive association between this infection and low HDL, elevated LDL, and cardiovascular disease—eradication lowered the risk of high LDL and low HDL *H. pylori* infection increased the risk of adverse cardiovascular events by 51% [70] *H. pylori* infection—a significant and independent predictor of dyslipidemia [71]	Korea → *H. pylori* had no effect on cardiovascular disease [72] Japan → no relationship between *H. pylori* infection and either stroke mortality risk or coronary heart disease [73]
**Insulin resistance, diabetes mellitus and obesity**	significantly higher prevalence of *H. pylori* infection in patients with diabetes [74] *H. pylori* prevalence—more increased in type 2 diabetes mellitus patients [75] *H. pylori*-associated inflammation + cytokines + hormonal imbalances = → association between this infection and diabetes mellitus [76] eradication → decreases the risk of diabetes [77] Song et al. → diabetic patients with *H. pylori* infection might require more aggressive eradication therapies [78] relationship between *H. pylori* infection and insulin resistance in normal-weight subjects [79] eradication rate lower in obese patients [80] *H. pylori* and high body mass index separately or not—risk factors for diabetes mellitus [81] Hamrah et al. → significant association between *H. pylori* infection and both diabetes mellitus and increased body mass index [82] Alzahrani et al.—a relationship only between *H. pylori* infection and increased body mass index [83] China → *H. pylori* is significantly associated with diabetes [84] Yang et al. → the severity of corpus gastritis associated to *H. pylori* infection was more severe in type 2 diabetes mellitus patients when compared to non-diabetic controls [85] *H. pylori*—a higher incidence of NAFLD [86] Polyzos et al.—NAFLD patients higher circulating levels of anti-*H. pylori* IgG [87] patients detected with *H. pylori* infection have a higher risk of NAFLD [88]	certain studies failed in identifying any association [89,90,91] obesity was not proven to enhance insulin resistance in the setting of *H. pylori* infection [92] Alzahrani et al.—no relationship between *H. pylori* infection and type 2 diabetes mellitus [83] China → *H. pylori* was not significantly associated with diabetes [93] Alzahrani et al. also failed in identifying any association between *H. pylori* seropositivity and risk of developing diabetes [94] pediatric patients → no significant association between diabetes and *H. pylori* infection [95] Japan and Korea → no relationship between *H. pylori* infection and NAFLD [96,97]
**Neurological conditions**	*H. pylori* might damage dopaminergic cells in central nervous system => Parkinson’s disease [98] Tan et al.—*H. pylori* might worsen Parkinson’s disease motor severity [99] eradication associated with an improvement of levodopa action, clinical symptoms, and life quality in Parkinson’s disease [20] host’s innate immunity—underlying link between Parkinson’s disease and *H. pylori* infection [100] multiple sclerosis → *H. pylori* infection more frequent in these patients [101] Long et al. [102]—an increased prevalence of *H. pylori* infection in multiple sclerosis Gerges et al.—a significantly higher *H. pylori* seropositivity in Egyptian multiple sclerosis patients [103] Baj et al.—both harmful and protective effects of *H. pylori* infection on multiple sclerosis [100] Alzheimer’s disease related to *H. pylori* [104] *H. pylori* might negatively impact Alzheimer’s disease development and clinical course [105] *H. pylori* infection—increased the risk of Alzheimer’s disease, while its eradication—improvement in Alzheimer’s disease symptoms [106,107] significantly higher levels of anti-*H. pylori* IgG antibodies in both serum and cerebrospinal fluid of Alzheimer’s disease [108] possible association between Alzheimer’s disease and gastrointestinal microbiota including *H. pylori* [109]	no association between bacterial eradication and Parkinson’s disease clinical outcome [110] Yaoa et al.—negative correlation between multiple sclerosis and *H. pylori* [111] Baj et al. also—both harmful and protective effects of *H. pylori* infection on multiple sclerosis [100]
**Dermatological conditions**	rosacea, also known as teleangiectasia → associated with *H. pylori* infection [112] eradication of *H. pylori* infection → improvement or total resolution of rosacea cutaneous lesions in 96.9% [113,114] chronic urticaria or itchy rash consisting of wheal-like lesions—associated with *H. pylori* infection [115,116] autoimmune bullous diseases consisting of pemphigus, pemphigoid, dermatitis herpetiformis, epidermolysis bullosa acquisita → associated with *H. pylori* infection [112]	other authors → no relationship between rosacea, chronic urticaria and *H. pylori* infection, still they pointed out a positive effect of eradication therapy on skin lesions [117] association between *H. pylori* infection and psoriasis or alopecia aerata remain contradictory [112]
**Ophthalmic disease**	relationship between open-angle glaucoma and *H. pylori* infection [118] prevalence of *H. pylori* infection higher in patients with this ophthalmic condition normalization of mean visual field parameters and intraocular pressure as a result of *H. pylori* eradication [119] central serous chorioretinopathy is associated with *H. pylori* infection [118] improvement in central serous chorioretinopathy and a reduction in the recurrence rate following *H. pylori* eradication [120,121]	-
**Autoimmune conditions**	B cells chronically stimulated with *H. pylori*-produced urease resulted in a production of autoantibodies, including IgM rheumatoid factor [122] the risk of rheumatoid arthritis and lupus erythematosus in the setting of *H. pylori* infection might be related to the virulence features of this bacterium [123] higher risk of systemic lupus erythematosus in *H. pylori* infected females <30 years [124] Sjögren syndrome → associated with *H. pylori* infection [125] *H. pylori* antibodies → significantly increased in patients with primary Sjögren syndrome [126] Increased prevalence of *H. pylori* infection in patients with systemic sclerosis [127]	the eradication of *H. pylori* infection effect on rheumatoid arthritis evolution remains also debatable since both positive [128,129] and null [130,131] findings were reported prevalence of *H. pylori* in patients with this autoimmune condition failed in proving a significant difference in prevalence rate when compared to healthy controls [15,132] *H. pylori* might represent a protective factor against systemic lupus erythematosus in African-American females [133] *H. pylori*—dichotomous role acting as both a trigger and a protector for systemic lupus erythematosus depending on age, race, and the affected organs [125] the benefit of *H. pylori* eradication in patients with this autoimmune condition remains controversial [134] other studies failed in identifying a significant difference regarding *H. pylori* prevalence in systemic sclerosis patients [135] all patients with systemic sclerosis were infected with more virulent *H. pylori* strains, especially those expressing CagA [135]

**Table 2:** Possible association between *H. pylori* and extra-gastrointestinal diseases—Meta-analysis-based evidence.

Type of Extra-Intestinal Manifestations		Authors, Year	Statements
**Hematological diseases**	Iron deficiency anemia	Qu et al., 2010 [26]	association between *H. pylori* and iron deficiency anemia in randomized controlled trials, eradication of *H. pylori* can improve hemoglobin and serum ferritin levels (not significantly)
		Hudak et al., 2017 [136]	*H. pylori*—decreased iron stores *H. pylori* eradication therapy, added to iron therapy → increased ferritin and hemoglobin levels
		Yuan et al., 2010 [138]	treatment of *H. pylori* infection could be effective in improving anemia and iron status in iron deficiency anemia
		Afsar et al., 2020 [139]	*H. pylori* infection is associated with increased risk of iron deficiency anemia in pregnancy
		Huang et al., 2010 [137]	*H pylori* eradication therapy combined with iron administration is more effective than iron administration alone for the treatment of iron deficiency anemia
	Purpura	Xiong et al., 2012 [46]	eradication therapy may reduce the recurrence of Schonlein Henoch purpura in children with *H. pylori* infection
		Kim et al., 2018 [140]	*H. pylori* eradication → therapeutic effect in patients with immune thrombocytopenic purpura
		Yu et al., 2011 [141]	eradication of *H. Pylori* increases platelet count in patients with immune thrombocytopenic purpura
**Growth**	Delayed growth in children	Wei et al., 2020 [142]	*H. pylori*-positive children were prone to delayed linear growth preventing and detecting *H. pylori* infection in children—critical to ensure normal growth and development during childhood
		Xu et al., 2022 [143]	*H. pylori* infection—associated with growth outcomes in children, mainly negative effect on children’s height-for-age
		Tang et al., 2021 [144]	*H. pylori* infection during pregnancy was significantly related to a higher rate of preeclampsia, fetal growth restriction, gestational diabetes mellitus, and hyperemesis gravidarum
		Zhan et al., 2019 [145]	*H. pylori* infection during pregnancy can increase the risk on adverse pregnancy outcomes (preeclampsia, fetal growth restriction, gestational diabetes mellitus, spontaneous abortion and birth defect)
**Cardiovascular diseases**	Atherosclerosis (AS)	Shi et al., 2022 [146]	*H. pylori* infection can promote the process of atherosclerosis in people <60 years and people without cardiovascular risk factors
		Keikha et al., 2022 [147]	positive relationship between *H. pylori* infection and atherosclerosis in the Iranian population, similar to Western countries
		Wang et al., 2021 [148]	significant association between *H. pylori* and subclinical atherosclerosis
	Stroke	Doheim et al., 2021 [149]	*H. pylori* infection—significantly associated with increased risk of stroke
		Yu et al., 2014 [150]	no strong association between *H. pylori* infection and stroke, neither in those with cytotoxin-associated gene-A-positive infection
	Systemic Arterial Hypertension	Huang et al., 2021 [76]	*H. pylori* infection is positively associated with hypertension
		Fang et al., 2022 [151]	*H. pylori* is a vital risk factor for hypertension
	Coronary heart disease	Rahmani et al., 2018 [152]	most of the patients acquired *H. pylori* infection during childhood personal hygiene promotion and preventive programs for Iranian children may have a role in reducing the risk of the infection and cardiovascular diseases
		Sun et al., 2016 [153]	*H. pylori* infection might increase the risk of coronary heart disease events, especially in early life, but weak
	Coronary instability	Franceschi et al., 2009 [154]	*H. pylori* strains might be critical to precipitate coronary instability mediated by antigen mimicry between CagA antigen and a protein contained in coronary atherosclerotic plaques
	Acute coronary syndrome	Fang et al., 2019 [155]	*H. pylori infection* was associated with an increased risk of acute coronary syndrome, especially in developing countries
	Arrhythmia	Yan et al., 2016 [156]	*H. pylori* infection was a risk factor for atrial fibrillation in Asia and Africa. a possible correlation between *H. pylori* infection and arrhythmia *H. pylori* eradication may decrease the occurrence of arrhythmia, especially in Asia and Africa
	Atrial fibrillation	Tetta et al., 2019 [157]	no significant correlation between *H. pylori* infection and atrial fibrillation
	Myocardial Infarction	Rahmani et al., 2017 [158]	*H. pylori*—associated with higher incidence of myocardial infarction in adults
**Metabolic syndrome**	Diabetes and Insulin resistance	Song et al., 2021 [78]	a higher risk of *H. pylori* eradication failure in type 2 diabetes mellitus (T2DM) *H. pylori* eradication could improve glycemic control in patients with T2DM
		Upala et al., 2017 [159]	*H. pylori* eradication does not improve insulin resistance, lipid metabolism parameters, or fasting blood glucose
		Mansori et al., 2020 [160]	high prevalence of *H. pylori* in patients with diabetes eradication of this bacterium should be considered in patients with diabetes
		Zhou et al., 2013 [161]	*H. pylori* infection increased in T2DM patients
		Shi et al., 2018 [162]	*H. pylori* infection was associated with the occurrence of proteinuria in T2DM patients
		Chen et al., 2019 [163]	correlation between *Helicobacter pylori* infection and glycated hemoglobin A levels in diabetes
		Azami et al., 2021 [164]	a possible relationship between metabolic syndrome, insulin resistance, and *H. pylori* infection
		Upala et al., 2016 [165]	*H. pylori* infection is positively associated with metabolic syndrome infection with *H. pylori* is associated with higher triglyceride, fasting blood glucose, body mass index (BMI), Homeostatic Model Assessment for Insulin Resistance (HOMA-IR), systolic blood pressure and lower high-density lipoprotein (HDL-C)
	Nonalcoholic Fatty Liver Disease (NAFLD)	Wijarnpreecha et al., 2018 [88]	a significantly increased risk of NAFLD among patients with *H. pylori* infection was demonstrated
	Lipid metabolism	Watanabe et al., 2021 [166]	*H. pylori* eradication increases the HDL-C levels
	Obesity	Baradaran et al., 2021 [167]	a positive correlation between the risk of *H. pylori* infection and the prevalence of obesity development
		Xu et al., 2019 [168]	China—obesity associated with *H. pylori* infection *H. pylori* infection may be one of the risk factors for obesity
**Neurological diseases**	Multiple Sclerosis	Yao et al., 2016 [111]	a significant lower prevalence of *H. pylori* infection in patients with multiple sclerosis *H. pylori* might be a protective factor for developing multiple sclerosis
		Jaruvongvanich et al., 2016 [169]	a significant lower prevalence of *H. pylori* infection in patients with multiple sclerosis *H. pylori* might be a protective factor for developing multiple sclerosis.
	Parkinson’s Disease (PD) and Alzheimer’s Disease (AD):	Fu et al., 2020 [105]	*H. pylori* has negative impact on the development of Parkinson and Alzheimer’s disease
		Dardiotis et al., 2018 [170]	higher prevalence of *H. pylori* infection in Parkinson disease patients suggesting that *H. pylori*
		Shen et al., 2017 [171]	*H. pylori* infection might be associated with the risk of Parkinson’s disease
		Liu et al., 2021 [172]	positive association between *H. pylori* infection and the risk of all-cause dementia, but not Alzheimer’s dementia
		Shindler-Itskovitch et al., 2016 [173]	*H. pylori* may play a role in the etiology of Alzheimer’s dementia
**Dermatological conditions**	Rosacea	Jørgensen et al., 2017 [174]	weak associations between rosacea and *H. pylori* infection effect of *H. pylori* therapy on rosacea symptoms → no statistical significance
	Chronic urticaria	Watanabe et al., 2021 [175]	antibiotics, especially those for *H. pylori* eradication, improved the remission rate and symptoms of chronic urticaria with few adverse events
		Gu et al., 2015 [176]	*H. pylori* infection is significantly, though weakly associated with an increased risk of chronic urticaria
**Ophthalmic disease**	Glaucoma	Zeng et al., 2015 [119]	significant association between *H. pylori* infection and open-angle glaucoma
	Glaucoma	Doulberis et al., 2020 [177]	*H. pylori* infection may be associated with glaucoma with null heterogeneity, as beyond histology, quantified by anti-*H. pylori* titers and increases with age
	Central serous chorioretinopathy	Liu et al., 2016 [178]	*H. pylori* infection → possible risk factor for central serous chorioretinopathy
**Autoimmune diseases**	Systemic lupus erythematosus, rheumatoid arthritis, autoimmune atrophy gastritis, and autoimmune pancreatitis	Youssefi et al., 2021 [123]	infection with more virulent strains of *H. pylori* cagA positive increase the risk of autoimmune diseases *H. pylori*—prevent the development of atrophic gastritis by stimulating inflammation in the gastric antrum.
	Sjögren syndrome	Chen et al., 2018 [179]	Significantly higher *H. pylori* infection rate in patients with SS
	Systemic sclerosis	Li et al., 2020 [180]	*H. pylori* infection may be associated with an increased risk of systemic sclerosis.
		Yong et al., 2018 [181]	Systemic sclerosis patients have an increased prior risk of *H. pylori* infection

## Data Availability

Not applicable.
